# AFM-based detection of glycocalyx degradation and endothelial stiffening in the db/db mouse model of diabetes

**DOI:** 10.1038/s41598-017-16179-7

**Published:** 2017-11-21

**Authors:** Marta Targosz-Korecka, Magdalena Jaglarz, Katarzyna E. Malek-Zietek, Aleksandra Gregorius, Agnieszka Zakrzewska, Barbara Sitek, Zenon Rajfur, Stefan Chlopicki, Marek Szymonski

**Affiliations:** 1 Center for Nanometer-scale Science and Advanced Materials, NANOSAM, Faculty of Physics, Astronomy and Applied Computer Science, Jagiellonian University, Łojasiewicza 11, 30-348 Krakow, Poland; 2 Jagiellonian Centre for Experimental Therapeutics, JCET, Jagiellonian University, Bobrzyńskiego 14, 30-348 Krakow, Poland; 3 Chair of Pharmacology, Jagiellonian University Medical College, Grzegórzecka 16, 31-531 Krakow, Poland; 4 Department of Biosystems Physics, Faculty of Physics, Astronomy and Applied Computer Science, Jagiellonian University, Łojasiewicza 11, 30-348 Krakow, Poland

## Abstract

Degradation of the glycocalyx and stiffening of endothelium are important pathophysiological components of endothelial dysfunction. However, to our knowledge, these events have not been investigated in tandem in experimental diabetes. Here, the mechanical properties of the glycocalyx and endothelium in *ex vivo* mouse aorta were determined simultaneously in indentation experiments with an atomic force microscope (AFM) for diabetic db/db and control db/+ mice at ages of 11–19 weeks. To analyze highly heterogeneous aorta samples, we developed a tailored classification procedure of indentation data based on a bi-layer brush model supplemented with Hertz model for quantification of nanomechanics of endothelial regions with and without the glycocalyx surface. In db/db mice, marked endothelial stiffening and reduced glycocalyx coverage were present already in 11-week-old mice and persisted in older animals. In contrast, reduction of the effective glycocalyx length was progressive and was most pronounced in 19-week-old db/db mice. The reduction of the glycocalyx length correlated with an increasing level of glycated haemoglobin and decreased endothelial NO production. In conclusion, AFM nanoindentation analysis revealed that stiffening of endothelial cells and diminished glycocalyx coverage occurred in early diabetes and were followed by the reduction of the glycocalyx length that correlated with diabetes progression.

## Introduction

The endothelium is a heterogeneous organ that maintains cardiovascular homeostasis^1–4^. From a morphological viewpoint, the endothelium consists of a monolayer of endothelial cells that lines the internal lumen of blood vessels. Importantly, the endothelium has a unique ability to convert mechanical stress induced by blood flow into biochemical responses, in particular, into the release of NO, the main vasodilator and an important vasoprotective molecule^5,6^. This unique feature of the endothelium is strongly related to the nanomechanical properties of the endothelial cells, in particular, to endothelial stiffness and the structural integrity of the glycocalyx layer. As shown by Fels *et al*.^7^, soft endothelial cells are more sensitive to shear stimulation than stiff cells and consequently produce more NO. NO influences vascular smooth muscle cells and causes vasodilation^8^. In endothelial dysfunction, endothelial cells stiffen, which impairs the vasodilation mechanism and leads to arterial stiffness and, consequently, hypertension^9,10^.

The endothelial glycocalyx has an important role in endothelial physiology. The glycocalyx is a brush-like surface layer of proteoglycans and glycoproteins that covers the luminal side of the endothelium^11^. It interacts directly with blood flow and plays important roles in endothelial mechanotransduction^12^ as well as in the modulation of vascular permeability^13^ and the regulation of hemostasis^14^.

Diabetes is associated with a number of macro and microvascular complications that are pathophysiologically linked with the development of endothelial dysfunction^15–17^. The high glucose concentration in blood plasma that accompanies the development of diabetes has a direct influence on endothelial cell metabolism^18^. As shown in both *in vitro* and *in vivo* biochemical experiments, hyperglycaemia induces increased production of reactive oxygen species (ROS)^19,20^. Overproduction of ROS leads to glycation processes, inflammation, and finally to endothelial dysfunction, as indicated by a pro-inflammatory, pro-thrombotic phenotype and impaired endothelial-dependent vasodilation^21^. Hyperglycaemia can also influence glycocalyx thickness and/or density and induce the collapse of the glycocalyx internal structure, with significant changes in the mechanical properties of this surface layer. In particular, loss of the endothelial glycocalyx during acute hyperglycaemia directly coincides with endothelial dysfunction^22^.

Force spectroscopy by nanoindentation with an AFM tip permits the study of the nanomechanical properties of the endothelium in both *in vitro* and *ex vivo* experiments^7,23–30^. For example, in our recent *in vitro* experiments^31^, force spectroscopy demonstrated that endothelial cells cultured under hyperglycaemic conditions became stiffer compared with cells grown under normoglycaemic conditions.

In this work, we performed *ex vivo* AFM nanoindentation experiments to simultaneously monitor structural parameters of the endothelial glycocalyx and elasticity of the endothelium from the aortas of C57BLKs/J-db/db (hereafter db/db) mice. The db/db mouse with a mutation in the leptin receptor gene is a well-established murine model of type 2 diabetes. Experiments were conducted in db/db mice at various ages to correlate the alterations in the structural parameters of glycocalyx and in the endothelial stiffness with diabetes progression and the development of endothelial dysfunction based on measurements of NO production.

To analyse the indentation data for the highly heterogeneous aorta sample a hybrid approach was implemented. All indentation curves were classified using the bi-layer brush model proposed by Sokolov *et al*.^32^. This model was originally designed for the study of cultured cells covered by a pericellular brush. In this work, the brush model allowed us to detect the endothelial glycocalyx layer in aorta samples and classify all indentation data into two main categories, i.e. into data recorded for endothelial regions with and without the glycocalyx surface layer. For regions of endothelium covered by the glycocalyx, the brush model was successively used for a quantitative analysis of the structural parameters of the glycocalyx surface layer (glycocalyx length and effective glycocalyx coverage) as well as for the determination of the apparent elastic modulus of the endothelial layer. For regions without glycocalyx, the apparent elastic modulus of the endothelium was derived using a straightforward application of the Hertz model to the initial part of the indentation curves.

## Analysis and Classification of indentation Curves

**Figure 1:**
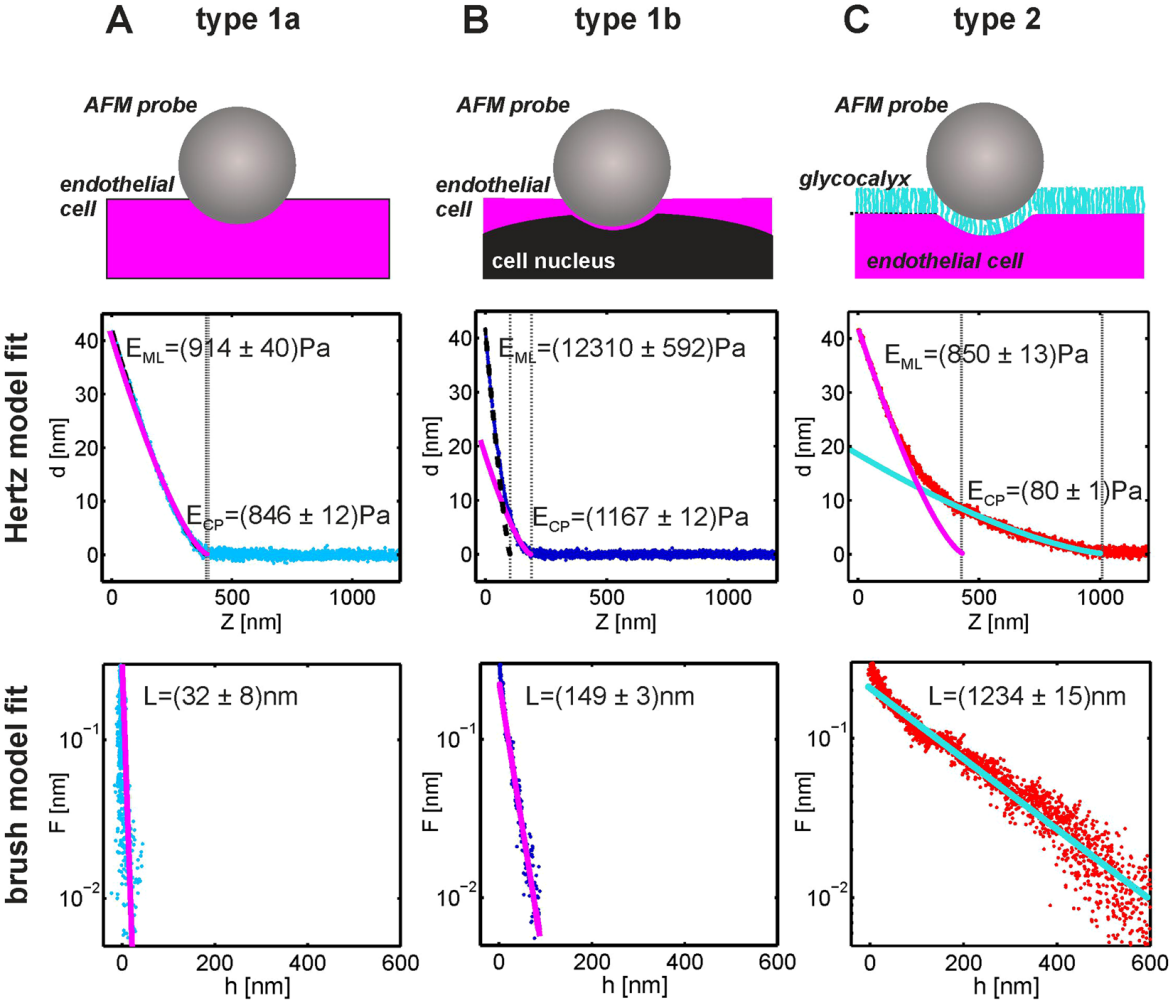
Examples of indentation data recorded in the experiment and method of analysis. (**A)** An indentation curve recorded for an endothelial cell body (referred to as a type 1a curve). (**B**) An indentation curve recorded for an endothelial cell body directly over the cell nucleus (type 1b curve). (**C**) Indentation curve recorded for an endothelial cell covered with the glycocalyx brush (type 2 curve). Top row: Schemes of indentation geometry. Middle row: Results of the Hertz model fit in the fragment of the contact point (CP) and in the fragment near the maximal load (ML). The dashed lines show the fit to the ML fragment, and solid lines show the fit to the CP fragment. Bottom row: *Ad hoc* brush model fit to the CP fragment of the curves.

The outermost layer of the aorta sample sensed by the AFM probe may correspond to either the glycocalyx surface layer or, in the case of a degraded glycocalyx, to the endothelial cell surface. The nanomechanical response of these layers must be analysed using different models. Moreover, the endothelial cells themselves may exhibit a depth-dependent response that is due to shell-like architecture of living cells and their contractility^33,34^. In general, the examined aorta patches exhibited large morphological and nanomechanical heterogeneity, as shown in Fig. 1 and S1 in the electronic Supplementary Information. This requires a dedicated method for the automatic analysis and classification of the large number of indentation curves recorded in the experiment.

### Hertz Model

The starting point of our analysis is based on Hertz contact mechanics^35^, which has been frequently applied for the determination of cell elasticity^36–39^. Application of contact theory provides a single parameter, the apparent elastic modulus, to describe the elastic properties of cells. Despite of controversies about the application of Hertz model to cell mechanics, the “apparent elastic modulus” is a useful parameter that provides a simple means for a direct comparison of data recorded in different experiments and/or different research groups^40^. It should be also emphasized that the Hertz model is inadequate for description of viscoelastic properties of cells^41,42^ and was used here only for analysis of the approaching part of the indentation curves.

In the Hertz model, the force *F*_*H*_ required to indent the cell surface by an indentation depth δ is given by the following expression:

1 $${F}_{H}(\delta )=\frac{16}{9}E\sqrt{R}{\delta }^{3/2},$$

where *R* is the effective probe radius and *E* is the effective elastic modulus of the endothelial cell (assuming the Poisson ratio of 0.5). In practice, the force that acts on the cantilever is determined from the cantilever deflection *d* through *F* = *kd*, where *k* is the spring constant of the cantilever. In our experimental geometry, the actual indentation can be calculated from the following combination of the piezo-scanner position *Z* and the cantilever deflection:

2 $$\delta ={Z}_{0}-Z-d,$$

where *Z*_0_ is the contact point (CP) position and *δ* and *d* are defined as non-negative quantities.

An example of a force indentation curve recorded on the endothelial layer from mouse aorta that is almost perfectly described by the Hertz model in equation (1) is shown in Fig. 1A. Such curves will be referred to as type 1a curves. The fit shown in Fig. 1A was performed by inserting equation (1) into equation (2) and by considering *E* and *Z*_0_ as fitting parameters.

Figure 1B presents an example of an indentation curve (referred to as type 1b) for which a fit of the Hertz model is impossible across the whole indentation range. We attribute this shape to the shell-like structure of the endothelial cells. We hypothesize that, at small indentation depths, the curve is dominated by the contribution from the cell cytoplasm and, at large indentation depths, by the mechanical response of the cell nuclei. One way, to describe such curves, could invoke generalization of equation (1) by introducing an indentation-dependent effective elastic modulus *E*(*δ*) and by performing a piece-wise analysis. The fit of the Hertz model to the curve in Fig. 1B in the range near the contact point (CP) gives a value of elastic modulus $\,{E}_{CP}=E(\delta \approx 0)$ that is similar to the value of the effective elastic modulus derived from the type 1a curve in Fig. 1A. The fit of the Hertz model near the maximal load (ML) gives a value of modulus $\,{E}_{ML}=E(\delta \approx {\delta }_{max})$ that is an order of magnitude larger (>10 kPa) and that may correspond to the elastic modulus of the cell nucleus.

Figure 1C presents yet another type of an indentation curve (referred to as type 2) for which a fit of the Hertz model with a single value of parameter *E* is not possible. Although equations (1) and (2) provide a reasonable approximation of type 2 curves at an indentation range close to the maximal load, the corresponding fit at CP range provides very small values of *E* indicating that the outer layer of the sample at this location is very soft. It is plausible, therefore, to imply that the “brush model”^32,43–46^, should be much more adequate for description of type 2 curves as described below.

### Brush Model

The glycocalyx is composed mainly of long glycoprotein chains and is usually described as a brush-like structure^47,48^. During squeezing of this layer, the forces have steric- or entropic-like character. An appropriate theory for their description is based on the Alexander-de Gennes theory of polymer brushes^49^. The force required to squeeze a brush of length *L* to a thickness *h* can be approximated as

3 $${F}_{B}(h)=50\,{{\rm{k}}}_{B}TR{N}^{3/2}L\,exp(-2\pi h/L),$$

where *k*_*B*_ is the Boltzmann constant, *T* is the temperature, and *N* is the brush grafting density. The model from equation (3) is valid for $0.2 < \frac{h}{L} < 0.9$. In an experiment, the thickness *h* can be identified as the separation between the AFM probe and the surface of the endothelium covered by the glycocalyx.

Sokolov *et al*.^32^ proposed a method for simultaneous derivation of the cell elastic modulus and determination of the glycocalyx parameters from a single indentation curve. They observed that in the case of a brush that covers the cell surface, equation (2) can be modified to the following form:

4 $$\delta -h={Z}_{0}-Z-d,$$

In the fragment of an indentation curve near the maximal load, one can assume that the brush is almost completely squeezed (h ≈ 0), and the values of cell elastic modulus can be extracted using the fit of the Hertz model from equation (2) that is limited to the data points in the region of maximum load. Next, the derived value of the cell elastic modulus can be used to calculate the forces due to the glycocalyx brush, and a fit in the form of equation (3) can be performed to determine the brush parameters.

Figure 1C shows the indentation curve recorded for an endothelial cell covered with glycocalyx. Such curves will be referred to as type 2 curves. The derived value of *E*_*ML*_ is comparable to values of *E*_*CP*_ derived from type 1 curves. The fit of the *F*_*B*_(*h*) formula for the brush force from Fig. 1C gives a value of brush length that is larger than 1 micron.

For a qualitative description of the “amount” of glycocalyx on the endothelium, the following product of brush length and grafting density can be used^50^:

5 $$n=NL.$$

This quantity can be interpreted as the total length of the brush molecules per unit area.

Forces due to the brush molecules at large h values are, in practice, difficult to measure. For example, for $h=L/2$, the exponential factor from Eq. (3) becomes $exp(-\pi )\approx 0.04$. Hence, for a maximal force due to the brush at the level of 0.25 nN at *h* = 0, the force at *h* = *L*/2 becomes 0.01 nN. For larger separations (*h* ~ *L*), the signal is covered by noise as evidenced in Fig. 1C.

Although Hertzian contact mechanics cannot be used directly for the description of steric-like force curves recorded on endothelium covered by glycocalyx (the apparent elastic modulus changes with indentation), several reports have defined the elastic or Young’s^44,51,52^ modulus, and other papers have used this quantity to characterize the glycocalyx^27,28^. Therefore, for sake of compatibility with previous studies of glycocalyx nanomechanics, the value of elastic modulus *E*_*CP*_ is also calculated for the type 2 curves recorded on the glycocalyx as shown in Fig. 1C. The value of the elastic modulus of the glycocalyx is, however, an order of magnitude smaller that the respective modulus of the endothelium.

### Automatic Analysis of Indentation Curves

**Figure 2:**
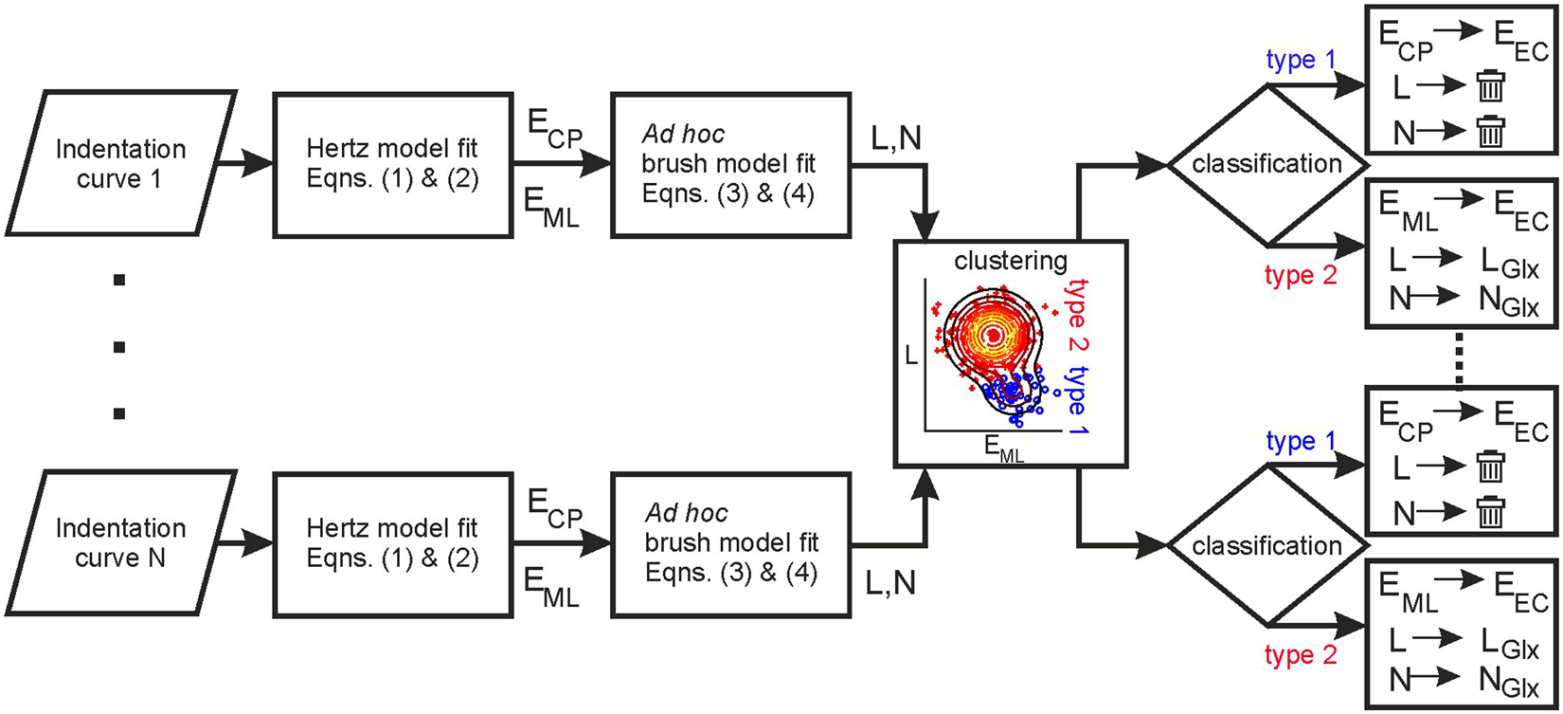
The flowchart of the data analysis process for automatic analysis and classification of indentation curves.

For a heterogeneous sample, *a priori* categorization of indentation curves and selection of the appropriate analysis method (Hertz vs. steric brush model) are impossible. Therefore, a multistep analysis for classification of the indentation curves and derivation of nanomechanical parameters has been implemented in this study. Since the nanomechanical parameters derived from individual indentation curves showed broad and sample-dependent distributions, the classification was based on clustering methods performed on large sub-populations of data. The analysis flowchart is shown in Fig. 2 and can be divided into the following steps.

*Step 1*. For each indentation curve, fits of the Hertz model from equations (1) and (2) to fragments of curves near the contact point (CP) and near the maximal load (ML) are performed. Hence, each indentation curve is parametrized by *E*_*CP*_ and *E*_*ML*_.

*Step 2*: Based on the value of *E*_*ML*_, a derivation of *F*(*h*) from Eqs (3) and (4) is performed for all curves. For type 2 curves, *F*(*h*) describes real forces due to the glycocalyx brush. For type 1 curves, *F*(*h*) provides a measure of the deviation of the indentation curves from a perfect Hertz model with the elastic modulus of *E*_*ML*_. The fitting parameter *L* gives a length scale on which this deviation can be observed. As shown in Fig. 1A–C, for type 2 curves, one obtains much smaller values of this parameter.

As a result of steps 1 and 2, each indentation curve is parameterized by *E*_*ML*_, *E*_*CP*_ and *L*, *N*. However, the physical meaning of those parameters is not imposed yet.

*Step 3*. After performing step 1 and step 2 for all indentation curves from a given sample, a classification of curves based on the clustering method is performed. For details, see the next paragraph.

*Step 4*. After classification, the fitting parameters are related to real nanomechanical parameters. For type 1 curves, *E*_*CP*_ is interpreted as the elastic modulus of the endothelium, while *L* and *N* are discarded. For type 2 curves, *E*_*ML*_ is interpreted as the elastic modulus of the endothelium, and *L* and *N* are interpreted as the thickness and grafting density of the glycocalyx, respectively.

### Classification of Indentation Curves

We used the data-classification procedure based on two independent parameters, E_ML_ and L. Due to a large spread in the values, these parameters were transformed to a logarithmic E_ML_-L scale and displayed on scatter plots. A bivariate Gaussian mixture was fit to the data points using a gmdistribution.fit function in Matlab. This function uses an iterative expectation maximization algorithm^53^ to find a Gaussian mixture model with k components (k = 2, in our case). For each dataset, the function was run 1000 times with different sets of random starting parameters. The maximum number of iteration in each run was set to 500. The solution with the largest likelihood was taken as the final estimate. A cluster function in Matlab was then used to assign data into the specific component of the Gaussian mixture distribution with the criterion of the largest posterior probability for the observation, weighted by the component probability. As a result, all indentation curves were classified as type 1 or type 2 curves. Please note, that in this data-classification procedure, there is no sharp distinction between type 1a and 1b curves.

**Figure 3:**
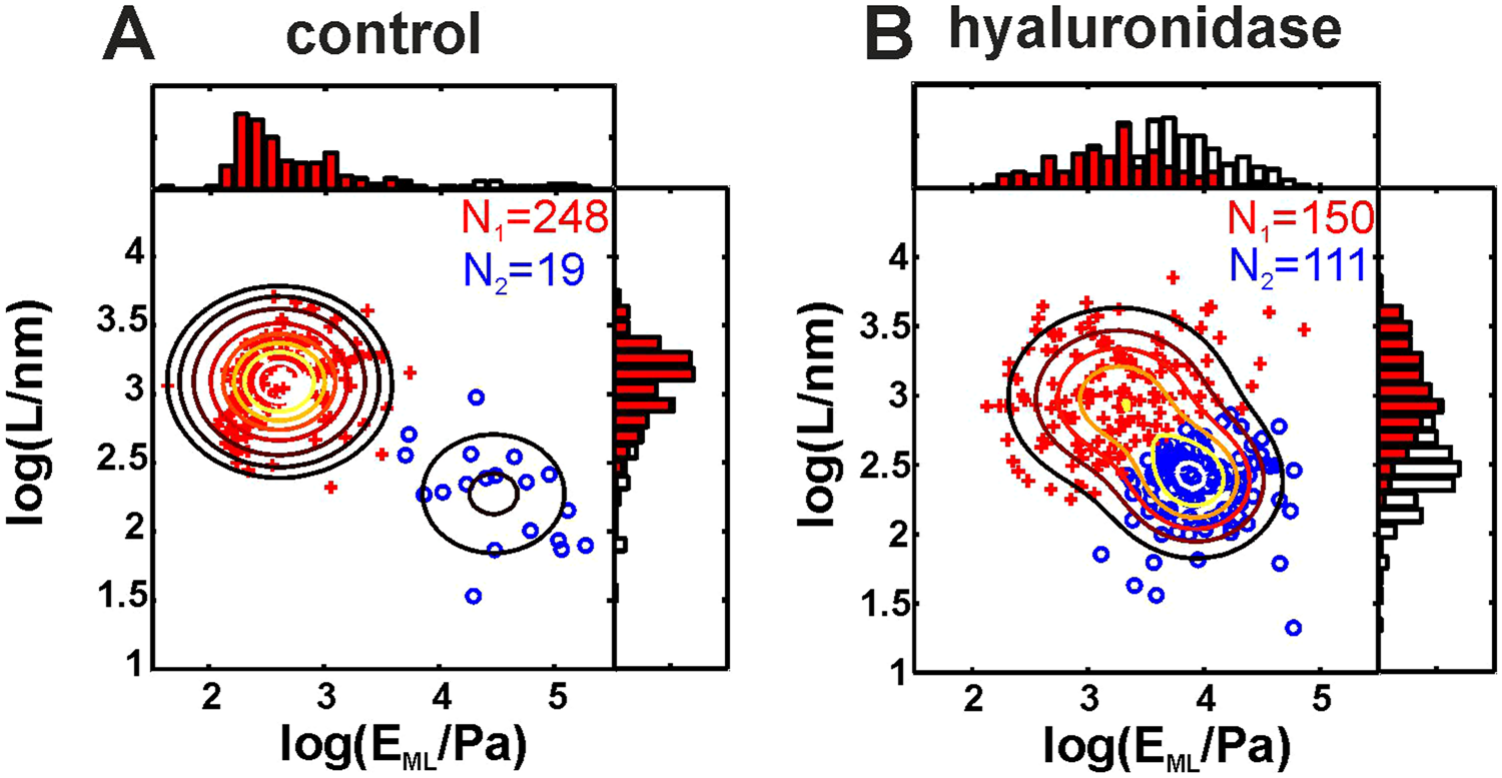
Validation of indentation data classification in a dedicated experiment with enzymatic degradation of the glycocalyx. (**A)** Data for the control aorta sample. **(B)** Data for the aorta sample after hyaluronidase incubation and partial glycocalyx degradation. Both panels show data as scatter plots of the elastic modulus at maximal load E_ML_ versus brush length L. The contour lines show Gaussian mixture distributions with two components fit to the data. The data points shown in blue (type 1 curves - without the glycocalyx) and red (type 2 curves - with the glycocalyx) were classified using an automatic clustering procedure. The histograms show the marginal distributions of E_ML_ and L (white bars – all data points, red bars – red data points). The glycocalyx degradation in (**B**) is manifested as an increase in the relative number of blue data points.

To validate the data-classification procedure, we performed a pilot experiment with enzymatic degradation^26^ of the glycocalyx from aorta extracted from C57BL/6 J mice. In one sample, the glycocalyx was intentionally partially digested by incubation with hyaluronidase solution (5U/ml, 2 h). The results of the data classification for the control sample and for the sample incubated with hyaluronidase are presented in Fig. 3. In both cases, the data cluster into two distinct components with similar mean values of E_ML_ and L but completely different point densities. For the control sample, the majority of the data points were classified as regions covered by the glycocalyx (red data points – type 2 curves), and only a small number of data points corresponded to regions without the glycocalyx. For the hyaluronidase-incubated sample, as expected, the relative number of curves classified as type 1 (blue curves, regions without glycocalyx) significantly increased. Accordingly, for each sample, the effective glycocalyx coverage can be defined as follows:

6 $${n}_{GLX}=\overline{n\,}\frac{{N}_{2}}{{N}_{1}+{N}_{2}},$$

where $\bar{n}$ is the average value of the local glycocalyx amount from equation (5) and the fraction gives the effective area of the sample with glycocalyx calculated from the number of curves classified as type 1 (*N*_1_) and type 2 (*N*_2_). In the example from Fig. 3, *n*_*GLX*_ is 102 ± 11 μm^−1^ for the control sample and 48 ± 9 μm^−1^ for the sample incubated with hyaluronidase.

## Results

### Progression of diabetes and development of endothelial dysfunction in db/db mice

**Figure 4:**
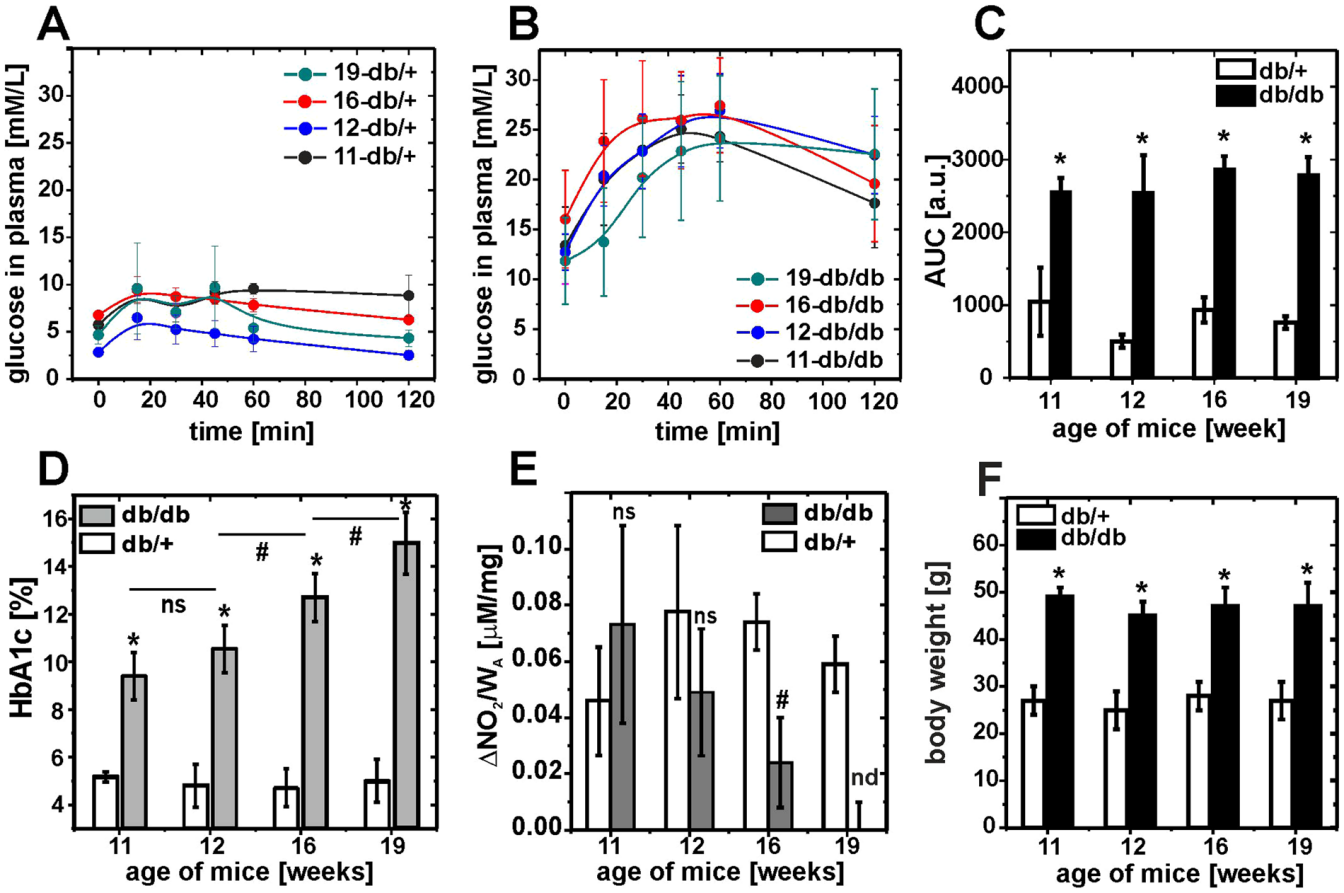
Diabetes progression and endothelial dysfunction in db/db mice. (**A,B)** The intraperitoneal glucose tolerance test (IPGTT) for control db/+ and db/db mice. **(C)** The area under the curve (AUC) in the IPGTT revealed a significant increase in the blood glucose concentration and indicated development of hyperglycaemia and an altered response to the glucose test consistent with insulin resistance. **(D)** Progressive increase in glycated haemoglobin A1C (HbA1C) content. **(E)** Progressive decline in NO production by the aorta (nd – non detectable). **(F)** Average mouse body weight. Values are shown as the mean ± SD. Statistics: n = 4; *p < 0.001, ^#^p < 0.01, ns- non significant.

To assess the progression of diabetes in db/db mice at the ages of 11–19 weeks, the intraperitoneal glucose tolerance test (IPGTT) was performed as shown in Fig. 4. As early as 11 weeks, db/db mice exhibited a significantly higher glucose plasma concentration and AUC than control db/+ mice, whereas older db/db mice did not display significantly higher values. By contrast, the plasma HbA1C concentration increased with age in db/db mice, reaching a value of 15 ± 2% in 19-week-old db/db mice (Fig. 4D). For db/+ mice, the concentration of glycated haemoglobin remained low and constant regardless of mouse age. As shown in Fig. 4E, NO production by the endothelium in the aorta measured as nitrite concentration progressively declined in aorta taken from 11- to 19-week-old db/db mice, with a significant decrease starting from week 16 confirming the phenotype of endothelial dysfunction.

### Experimental approach to study the nanomechanical properties of glycocalyx and endothelium in *ex vivo* aorta

**Figure 5:**
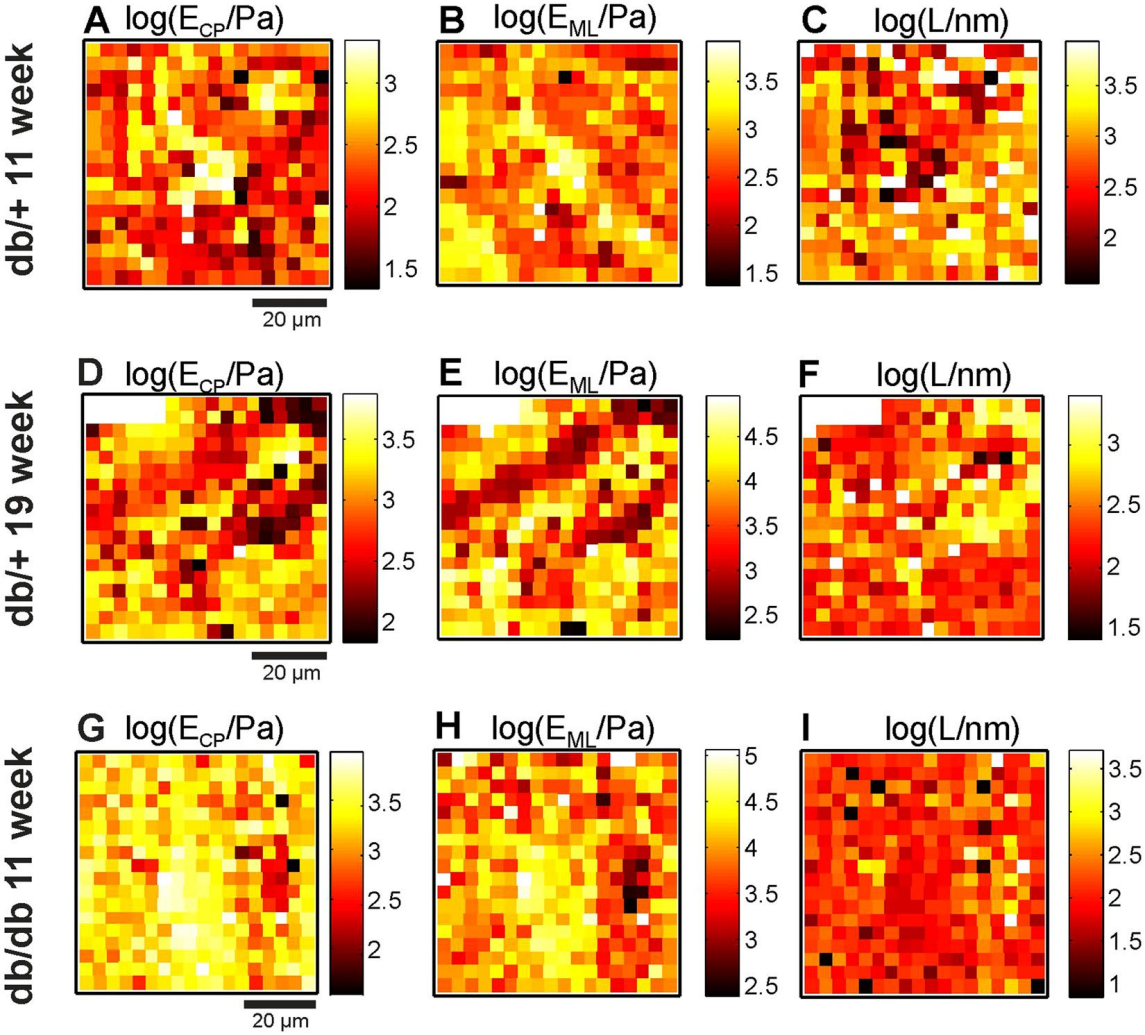
AFM nanoindentation data measured for endothelia from db/db and db/+ mice aortas. Upper row: Data for an 11-week-old control db/+ mouse. Middle row: Data for a 19-week-old control db/+ mouse. Bottom row: Data for an 11-week-old diabetic db/db mouse. **(A,D,G)** Values of the apparent elastic modulus derived from fragments of indentation curves near the contact point E_CP_. (**B,E,H)** Values of the apparent elastic modulus derived from fragments of indentation curves near the maximum load *E*_*ML*_. **(C,F,I)** Values of the L parameter. A logarithmic (base 10) scale is used for the colour scale. In all images, large spatial and mechanical sample heterogeneity can be observed.

Selected examples of AFM nanoindentation data measured for *en face* prepared aorta samples are presented in Fig. 5. Data are shown for the youngest and oldest db/+ control mice at ages of 11 and 19 weeks and for an 11-week-old diabetic db/db mouse. As described in the Methods, in the initial analysis steps, all measured indentation curves were *ad hoc* parametrized by the elastic modulus at small indentation depths *E*_*CP*_, the elastic modulus at large indentation depths *E*_*ML*_, and the “brush” length *L*. For direct visualization of sample morphology, those values are presented in the form of spatial maps.

**Figure 6:**
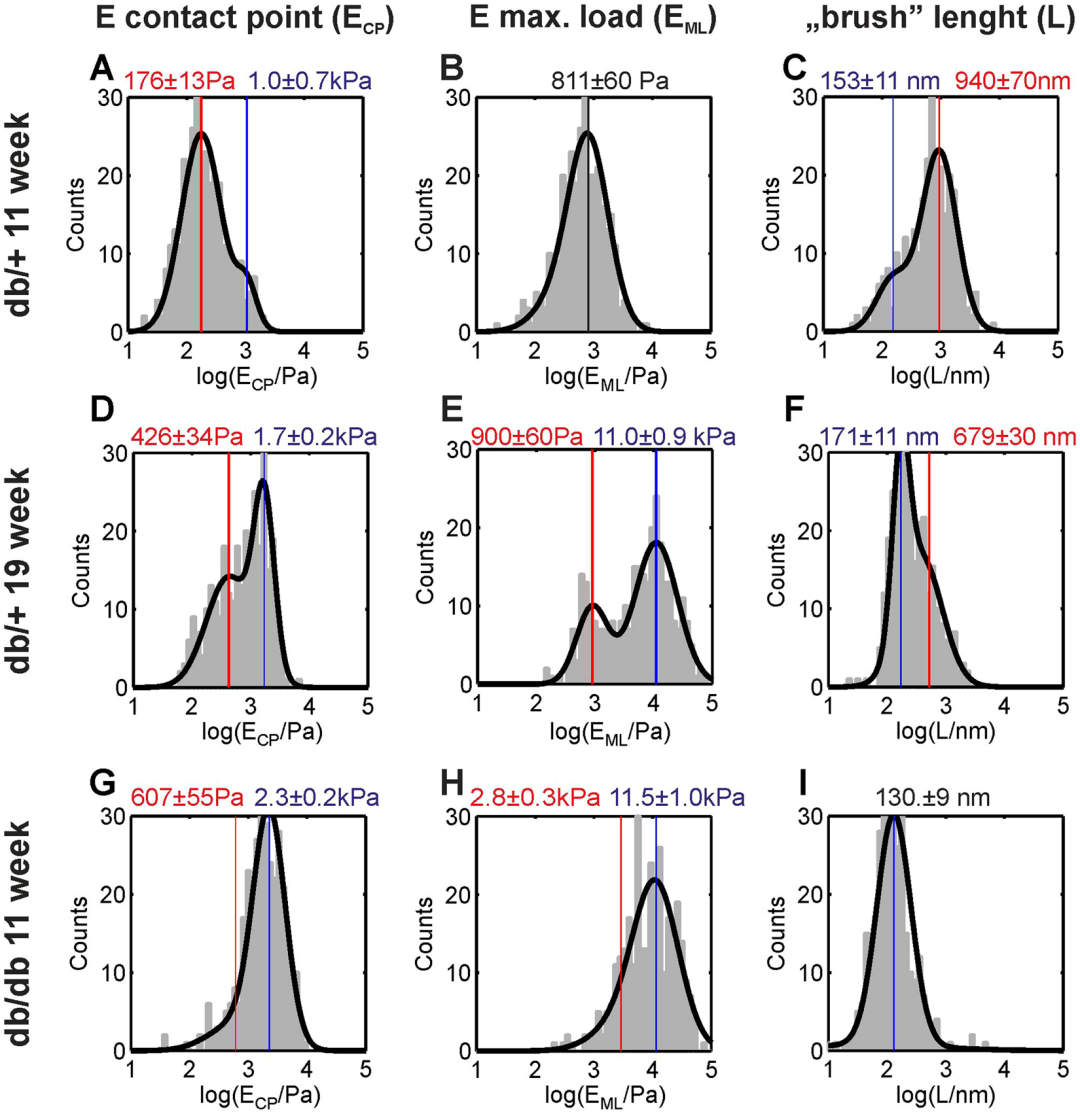
Histograms of the mechanical parameters derived from the maps shown in Fig. 5. (**A**–**C**) Histograms of the apparent elastic modulus E_CP_ derived for small indentation depths near the contact point. **(D–F)** Histograms of the apparent elastic modulus E_ML_ for large indentation depths near the maximal load. **(G–I)**. Histograms of the “brush” length L. In each histogram, depending on mouse age and type, two components with varying amplitudes can be clearly observed. The bimodal character of the data indicates the presence of two different sample regions (with and without the glycocalyx). Vertical lines and the corresponding text labels show the mean values of the Gaussian components.

In addition, for quantitative analysis, histograms of the nanomechanical parameters derived from the two-dimensional maps are presented in Fig. 6. Due to large sample heterogeneity, all values were transformed to a base 10 logarithmic scale.

The data from Figs 5 and 6 reveal important effects observed in this work. First, the maps in Fig. 5 indicate large sample heterogeneity and the presence of several structural features. Second, some histograms in Fig. 6 have a bi-modal character that is related to the presence of two distinct types of sample regions with very different mechanical properties. The bi-modal distribution of all mechanical parameters is most clearly visible in Fig. 6D–F, i.e., for data recorded for a 19-week-old db/+ mouse. For 11-week-old db/+ and db/db mice, all histograms are dominated by a single peak, but for the 11-week-old db/db mice, the bi-modal character of the distribution is still clearly visible.

The spatial map recorded for 11-week-old db/+ mouse is almost entirely dominated by “soft” regions (E_CP_ ≈ 176 Pa and E_ML_ ≈ 811 Pa), for which one can observe a long brush with L ≈ 940 nm. These regions are identified as endothelial areas covered by glycocalyx brush. For the older 19-week-old db/+ control mouse, in addition to “soft” regions, “hard” regions with E_CP_ ≈ 1.7 kPa and E_ML_ ≈ 11 kPa and with short apparent brush length can be identified. These regions are identified as regions without a glycocalyx. In the data recorded for the diabetic 11-week-old db/db mice, “hard” regions dominate the entire imaged surface of the sample.

**Figure 7:**
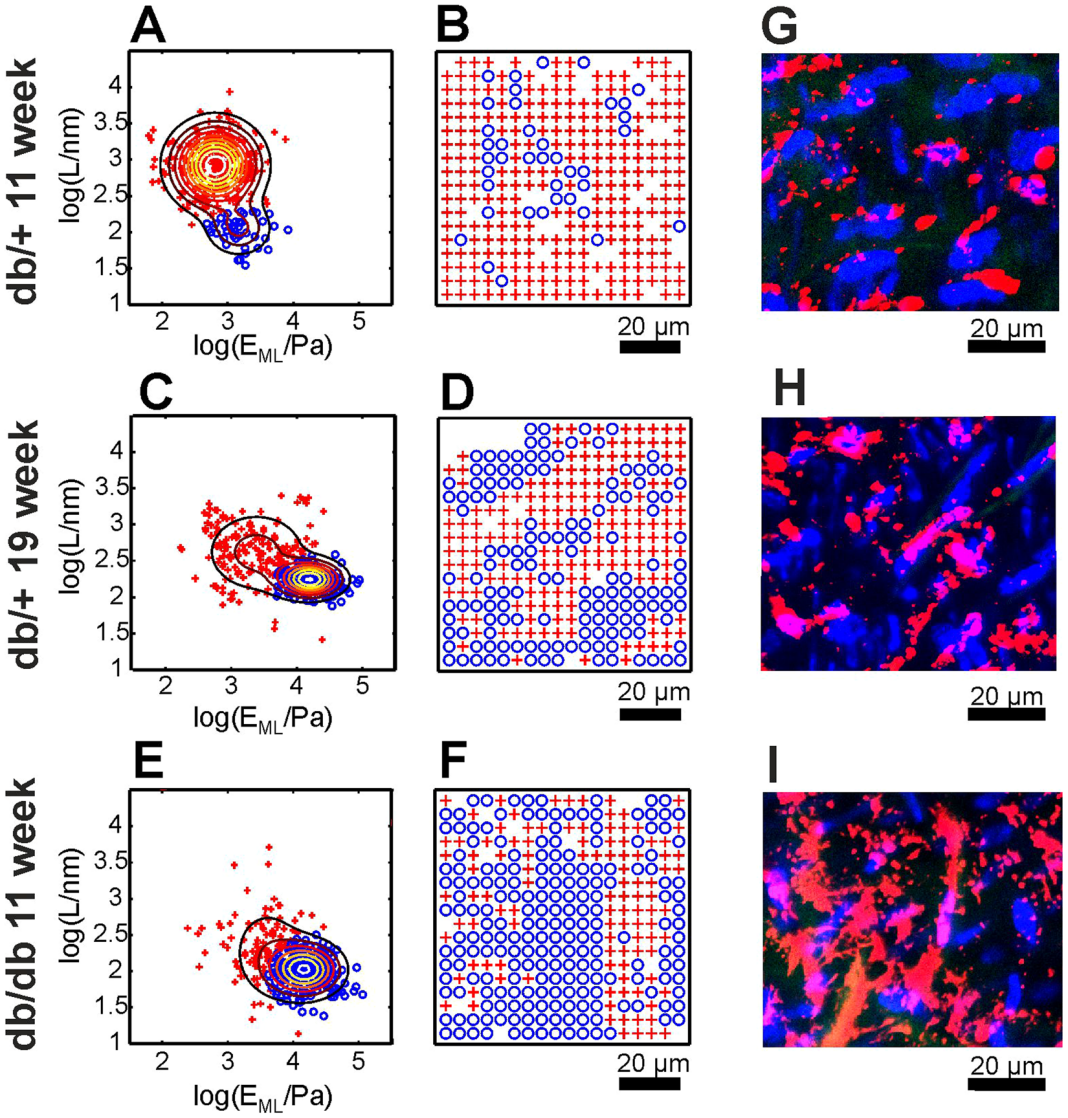
Data classification and automatic detection of the glycocalyx on endothelial cells from db/db and db/+ mice. The scatter plots **(A,C,E)** were constructed using the values of the apparent elastic modulus E_ML_ and values of brush length L presented in Fig. 6. The contour lines show Gaussian mixture distributions with two components fit to the data. The data points shown using red crosses and blue circles were classified using an automatic clustering procedure. The data classification results are presented in the form of spatial maps in **(B,D,F)**. Red crosses indicate endothelium covered with the glycocalyx (type 2 curves), and blue circles indicate endothelium without the glycocalyx brush (type 1 curves). For comparison, **(G–I)** show immunofluorescence staining of *en face* aorta samples. Blue: cell nuclei (staining by Hoechst). Red: von Willebrand factor. Green: autofluorescence of elastin.

To distinguish between regions with and without a glycocalyx, automated data classification was performed, as described in the Methods. The classification was based on scatter plots of *E*_*ML*_ and *L*, which are presented in Fig. 7(A,C,E). In all scatter plots, the data are grouped into two distinct components. As schematically visualized in Fig. 7G, the blue data points are classified as type 1 curves acquired on the parts of endothelial cells without a glycocalyx, and the red data points are classified as type 2 curves measured for the regions covered with a glycocalyx. Importantly, for 11-week-old diabetic db/db mice and, partially, for control 19-week-old db/+ mice, one can observe the effect of glycocalyx degradation. The glycocalyx degradation is manifested as an increase in the relative number of blue data points (as a reference, see the enzymatic degradation of glycocalyx from Fig. 3).

In Fig. 7(B,D,F), the data classification results are presented in the form of spatial maps that are commensurate with the spatial maps in Fig. 5. Instead of presenting the values of the mechanical parameters derived from indentation curves, the maps in Fig. 7H–J show the results of the classification procedure. Both the red and blue points form well-resolved regions that correspond to endothelium covered by glycocalyx or without the glycocalyx brush. Note that an incorrect classification would result in a quasi-random pattern. The comparison of data for 11-week-old db/db and db/+ mice indicates strong degradation of the glycocalyx and a change in its spatial distribution. Thus, the glycocalyx is strongly degraded in diabetic mice.

To verify the origin of the specific distribution of the glycocalyx, immunofluorescence imaging was performed. Examples of recorded images are shown in Fig. 7(H,I,J). Images were recorded for the same samples but at different locations than the AFM data. Previous work demonstrated marked heterogeneity of the glycocalyx composition between cell junctions and nuclear regions as well as correlation of glycocalyx degradation with the exocytosis of Weibel-Palade bodies, which are storage granules for the endothelium-specific von Willebrand factor (vWF). Therefore, Fig. 7(H,I,J) present the merged fluorescence signal from endothelial cell nuclei (blue) and from vWF (red).

### Evaluation of the nanomechanical properties of glycocalyx and the endothelium in the db/db mouse aorta in the course of diabetes progression

**Figure 8:**
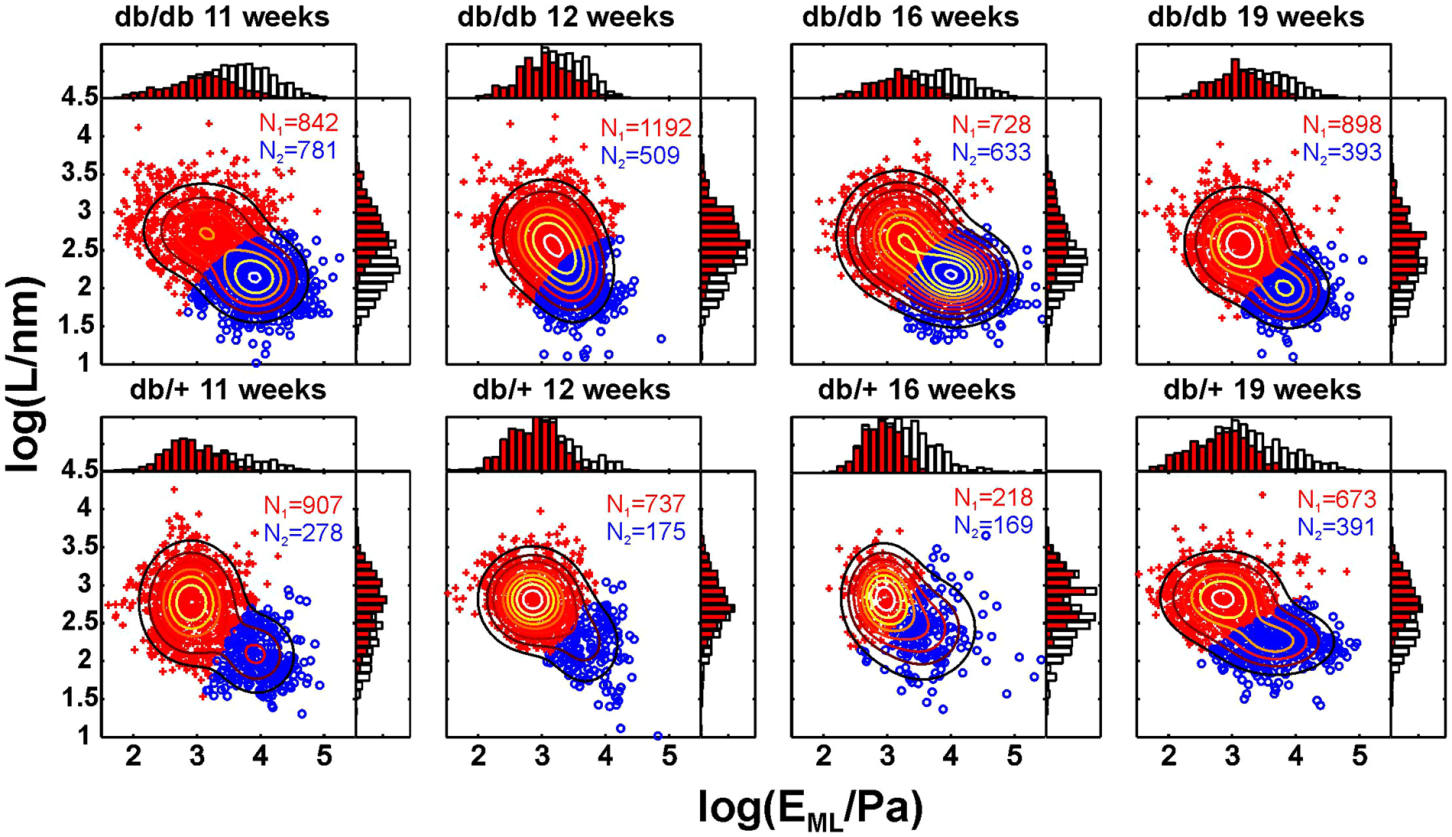
Complete experimental dataset and its classification for diabetes (db/db) and control (db/+) mice at different ages. Each panel shows data in the form of a scatter plot of the elastic modulus at maximal load E_ML_ versus brush length L. The contour lines show Gaussian mixture distributions with two components fit to the data. Data points shown in red (with the glycocalyx) and blue (without the glycocalyx) were classified using an automatic clustering procedure. The histograms show the marginal distributions of E_ML_ and L (white bars – all data points, red bars – red data points). Numeric labels show numbers of data points with the glycocalyx (red) and without the glycocalyx (blue) detection.

For a quantitative demonstration of glycocalyx degradation and endothelial stiffening in diabetes progression, the classification of nanoindentation data from the previous section was used to analyse the entire dataset recorded in the experiment. Figure 8 presents the whole experimental dataset (approx. 9,500 nanoindentation curves) in the form of scatter plots for aorta samples extracted from mice at different ages.

The upper row of scatter plots corresponds to data acquired for diabetic db/db mice, and the lower row corresponds to control db/+ mice. The numbers of curves that were classified as covered by glycocalyx or without glycocalyx are shown as red/blue text labels in each plot. Since each scatter plot in Fig. 8 corresponds to many random locations in aortas from different mice, the data present a much larger spread compared with the scatter plots in Fig. 7.

**Figure 9:**
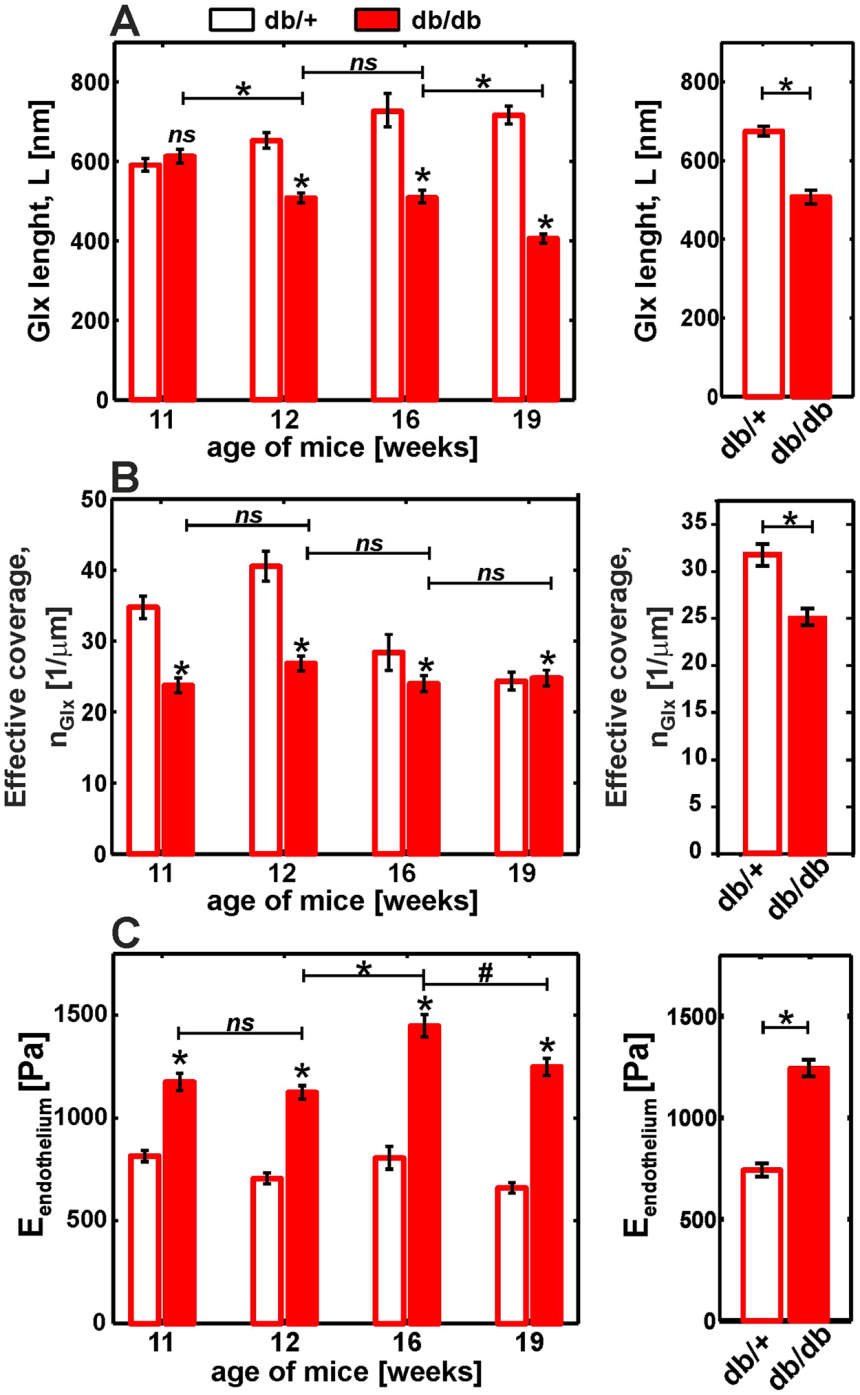
Detection of glycocalyx degradation and endothelial stiffening by AFM nanoindentation experiments in *ex vivo* mouse aorta. (**A**) Glycocalyx length. (**B**) Effective glycocalyx coverage. (**C**) Endothelium elastic modulus derived for type 2 curves (for full data see Figure S3 in Supplementary Information). The plots on the right side show the age-averaged values. Statistical significance was tested by two-way ANOVA followed by multiple-comparison Bonferroni tests. *p < 0.0001. (ns) non-significant.

As described in the Methods (Fig. 2), the classification allowed the parameters E_ML,_ E_CP_, L and N to be ascribed to the real nanomechanical parameters of the endothelial layer. The derived mean values of the glycocalyx brush length and effective glycocalyx coverage (equation 6) are presented in Fig. 9A and in Fig. 9B, respectively. The values of the endothelial elastic modulus are shown in Fig. 9C for type 2 curves. For the expanded analysis of the full dataset and a comparison of elastic moduli derived from type 1 and type 2 curves see Figure S2 in electronic Supplementary Information. For endothelium regions covered by the glycocalyx (type 2 curves) the apparent elastic modulus of the endothelial layer was derived from the brush model and for regions without glycocalyx (type 1 curves) using a straightforward application of the Hertz model to the initial part of the indentation curves.

## Discussion

In the present study, AFM indentation was used for *ex vivo* evaluation of the nanomechanics of the endothelium from db/db mouse aorta. We observed a large heterogeneity of the aorta samples, which made the analysis of AFM experiments challenging. The application of an automatic method for the analysis and classification of indentation curves allowed endothelial areas with or without a glycocalyx surface layer to be distinguished and the selection of an appropriate method for data analysis. We were thus able to evaluate the mechanical parameters of the glycocalyx surface layer and endothelium in diabetes progression and correlate them with biochemical markers of endothelial dysfunction.

### Properties of the glycocalyx surface layer

The mechanical parameters of the endothelial glycocalyx were derived after the classification procedure from type 2 curves. For the control db/+ mice, the average of results in Fig. 9A, gives the mean thickness of the glycocalyx brush of L = (674 ± 13) nm. However, in all samples, a very broad distribution of brush lengths from less than 300 nm to greater than 3 µm was observed (see Figs 3, 6 and 8). These values are in an excellent agreement with a recent accurate determination of the endothelial glycocalyx layer in an on-chip experiment^54^, which gave a value of 670 ± 200 nm and in qualitative agreement with confocal imaging of the glycocalyx from mouse aorta, which gave a mean glycocalyx thickness of 2.1 µm^48^. The mean value of the glycocalyx thickness reported in this work is, however, much higher than values reported in previous *ex vivo* AFM nanoindentation studies, which gave values of either 266 ± 12 nm^26^ or less than 100 nm^55^. We suppose that an underestimation of the glycocalyx thickness could be due to an oversimplified calculation of the mean value from both type 1 and type 2 curves. The apparent elastic modulus of the endothelial glycocalyx in db/+ mouse aorta has a value between 100 Pa and 500 Pa (see Fig. 6). These values are similar to the values reported in *in vitro* experiments^27,28^. For db/db mice, we observed a reduction in glycocalyx length to an age-averaged value of 507 ± 16 nm. Previously, a similar effect was observed *in vitro* for inflammation-induced damage in sepsis^26^.

### Spatial distribution of the glycocalyx

We observed marked heterogeneity of the glycocalyx layer in control db/+ mice. As shown in Fig. 7A and C, we observed areas with quite uniform coverage of the glycocalyx as well as endothelial regions without the glycocalyx surface layer. As shown in Fig. 7B, the redistribution of the glycocalyx was also observed in db/+ mice. The heterogeneity of the glycocalyx layer at the cellular scale is related to deficiency of the glycocalyx at the nuclear parts of the cells. The positions of the cell nuclei in Fig. 5B,E,H can be identified basing on the high value of E_ML_. For type 1 curves, the derived value of the elastic modulus at a large indentation depth E_ML_ has a mean value of 6150 ± 190 Pa. This value is averaged over all curves recorded in the experiment. However, in the examples from Fig. 6, values exceeding 10 kPa were also observed. We attribute such high values of the elastic modulus to the contribution from cell nuclei. Callie *et al*.^56^ observed an elastic modulus of the endothelial nucleus of ~5 kPa for nuclei in the cell and ~8 kPa for isolated nuclei. The phenomenon of non-uniform spatial distribution and remodelling of the glycocalyx (from and into nuclear and junctional parts of the cells) has been previously investigated by immunofluorescence and AFM imaging^27,28,57–59^. The elongated shape of the areas without the glycocalyx layer that is clearly visible in Fig. 5D–F is a consequence of the specific spatial distribution of endothelial cell nuclei, as is also evident in the fluorescence image in Fig. 7, and may result from alignment of the cells along the blood flow direction^58^. Also, please note that such high values of elastic modulus could not be detected for type 2 curves. For type 1 curves, the glycocalyx brush layer is very thin or not existing at all. Hence the indentation with the same load force could sample deeper endothelium body layers than in case of a very thick brush. The high values of the elastic modulus may be also caused by the presence of gaps in the endothelial layer. However, fluorescence imaging revealed a quite uniform layer on the endothelial cells as exemplified in Fig. 7G,H,I.

In addition to local fluctuations in the distribution of the glycocalyx at the cell surface, we observed spatial heterogeneity of the glycocalyx at larger length scales. This effect was most visible in the data for db/db mice in Fig. 7F but was also present in the db/+ data. In general, endothelial cells exhibit heterogeneity, which may exist even between neighbouring endothelial cells exposed to the same extracellular environment. For example, very recently, it was shown that the endothelium-specific vWF was expressed in a mosaic pattern in the aorta^60^. Also very recently, it was demonstrated that a patchy-like degradation of the glycocalyx may be related to the exocytosis of Weibel-Palade bodies, which are the source of vWF^59^. Our results from Fig. 7 appear to be consistent with those findings and reveal a similar patchy-like distribution of the glycocalyx and vWF.

### Mechanical properties of the endothelium

For the control db/+ mice, the age-averaged value of the endothelium elastic modulus was E_EC_ = 862 ± 16 Pa. This value is in agreement with the modulus value of the cytoplasm in endothelial cells measured in a glass microplate compression experiment^56^ and with our previous *in vitro* studies^61^. For db/db mice, we observed a significant increase in the age-averaged value of the endothelium elastic modulus to 1449 ± 18 Pa. Our recent *in vitro* study revealed similar changes for EA.hy926 endothelial cells in response to chronic hyperglycaemia^31^.

### Changes of mechanical parameters of the endothelium and endothelial glycocalyx in diabetes progression and their relation with biochemical markers

As shown in Fig. 4C, IPGTT revealed a significant increase in blood glucose concentration and steady hyperglycaemia in db/db mice. Similarly, the endothelium elastic modulus (Figs 9C and S2 in electronic Supplementary information) and effective coverage (Fig. 9B) were already altered in 11-week-old db/db mice and these alterations remained at approximately similar level in older db/db mice.

In contrast, glycocalyx length (Fig. 9A) was not altered in 11-week-old db/db mice in comparison with db+ mice, but it was gradually diminished in older db/db animals. Interestingly, the changes in the glycocalyx length correlated with the progression of diabetes, as assessed by monitoring the HbA1 concentration (Fig. 4D), and progressive endothelial dysfunction, as evidenced by the decrease in NO production (Fig. 4E). These results suggest that the diabetes-induced endothelial stiffening and loss of glycocalyx coverage are both mechanistically linked to early diabetes, which is characterized by pronounced hyperglycaemia and insulin resistance. However, the shrinking of the glycocalyx length in areas with preserved endothelial glycocalyx is a consequence of prolonged diabetes manifested by increased HbA1.

Interestingly, progressive reduction of the glycocalyx length totalled nearly 45% in 19-week-old db/db mice compared with db/+ mice. Previous studies have shown that hyperglycaemic conditions in humans and mice with type 1 and type 2 diabetes are associated with reduced glycocalyx dimensions and/or an increased release of the glycocalyx constituents in plasma^62,63^. In turn, in endotoxaemic mice, an AFM nanoindentation study^26^ reported a 50% glycocalyx length reduction, similar to the extent of glycocalyx injury we observed in diabetes. Importantly, our results demonstrate that the two aspects of glycocalyx injury, i.e. loss of glycocalyx coverage and reduction in glycocalyx length seem to be mechanistically unrelated. Interestingly, Zeng *et al*.^57^ showed that structural changes in the glycocalyx layer upon enzymatic treatment are evident as both a decrease in glycocalyx coverage and a reduction of the glycocalyx thickness. Obviously, the mechanisms of enzymatic glycocalyx disruption are different to the changes in diabetes induced by prolonged hyperglycaemia and its consequences. In our experiments in diabetic mice, the changes in glycocalyx coverage were pronounced at earlier stages and were associated with increased endothelial stiffness, whereas the reduction in glycocalyx thickness occurred gradually along the progression to advanced diabetes.

In conclusion, we have shown that the method proposed by Sokolov *et al*.^32^ is a valuable tool for the analysis of AFM nanoindentation data from *ex vivo* vascular preparations. However, given the heterogeneous nature of vascular preparations, AFM nanoindentation must be not applied directly but in combination with a classification procedure such as that proposed and validated in this study. The combination of AFM nanoindentation-based detection of glycocalyx degradation and endothelial stiffening with the approach to data analysis presented in this work provides new insights into the nanomechanics of endothelial dysfunction in a murine model of type 2 diabetes. We demonstrated that diabetes-induced endothelial stiffening and loss of glycocalyx coverage were both present in early diabetes and remained similar in advanced stages of diabetes in older db/db mice. However, the shrinking of the glycocalyx length in areas with preserved endothelial glycocalyx was progressive and correlated with progression of diabetes (assessed based on blood HbA1 concentration) as well as with progressive impairment of endothelial function (NO production). Accordingly, the diabetes-induced endothelial stiffening accompanied by the loss of glycocalyx coverage represents an early feature of endothelial dysfunction in diabetes. On the other hand, the shrinkage of the glycocalyx length may be regarded as the manifestation of the advanced endothelial pathology in diabetes. Although endothelial stiffening accompanied by the loss of glycocalyx coverage and shrinkage of the glycocalyx length may be interlinked, temporal dissociation of their occurrence along the progression of diabetes may suggest different mechanisms involved.

## Methods

### C57BL mice

C57BLKs/J-db/db male mice and age-matched C57BLKs/J-db/+ mice and C57BL/6 J mice were purchased from Charles River Laboratories. Animals were housed in specific pathogen-free conditions (SPF) and fed a standard laboratory diet and water ad libitum. All experimental procedures used in the present study were conducted according to the Guidelines for Animal Care and Treatment of the European Communities and the Guide for the Care and Use of Laboratory Animals published by the US National Institutes of Health (NIH Publication No. 85-23, revised 1996). All procedures involving animals were approved by the Local Bioethics Committee in Krakow, Poland, and were conducted in accordance with institutional guidelines. To evaluate the effects of diabetes progression on endothelial stiffening and glycocalyx properties, the db/db and db/+ mice were studied at the ages of 11, 12, 16 and 19 weeks. For each age group, aorta samples were harvested from four db/db mice and four db/+ mice (n = 4). Mice were anaesthetized with ketamine and xylazine, and their aortas were isolated.

### Blood HbA1c and IPGTT

Blood was collected from the right ventricle from anaesthetized animals with a syringe containing nadroparine (final concentration: 10 U/ml). HbA1c and total haemoglobin concentrations were measured in full blood using a biochemical analyser (ABX Pentra 400, HORIBA), and the ratio was given as a percentage of HbA1c. For the glucose tolerance test, mice were fasted for 4 h and then injected intraperitoneally with glucose solution at 2 g/kg of body weight (Sigma-Aldrich, St. Louis, MO, USA). Blood was collected from the tail vein before (0 min) and at 15, 30, 45, 60 and 120 min after glucose administration for plasma glucose measurements (Pentra 400, Horiba, Kyoto, Japan). The IPGTT results were expressed as the area under the curve (AUC) of blood glucose concentration.

### Analysis of NO production by the aorta

Basal NO production by the aorta was estimated using measurements of nitrite, which is a primary stable product of nitric oxide oxidation and thus was considered appropriate for the estimation of NO synthesis by the aortic endothelium. Segments from the aortic arch were longitudinally opened, placed in a 96-well plate with the endothelium facing up, and incubated covered for one hour in 120 µl of K-H buffer at 37 °C on a rocker. The nitrite concentration was measured with an ENO-20 NOx Analyzer (Eicom Corp., Kyoto, Japan) based on a liquid chromatography method with post-column derivatization using Griess reagent. The limit of detection was approximately 10 nM nitrite. Multiple blank samples (without aortic rings) were used to monitor nitrite contamination in the buffer and/or laboratory atmosphere in every set of experiments. The averaged blank signal for a given set of experiments was subtracted as a background signal. Samples were kept on ice and analysed directly after experiments. Nitrite concentration was expressed as µM/mg of wet weight of aortic rings.

### Aorta sample preparation

The aorta samples were resected from a descending thoracic fragment of the aorta. To prepare *en face* samples of aortas, a protocol described by Wiesinger *et al*.^26^ was used. After harvesting, the aorta samples were immediately transferred to PBS buffer, gently rinsed and cleaned from surrounding tissue. Next, the aorta was cut into small rings, and then a single ring was cut into small patches to expose the inner wall of the aorta. The patches of the aorta were gently transferred onto a glass coverslip coated with Cell-Tak® (BD Biosciences, Bedford, MA, USA). The patch of the aorta was glued to the glass leaving the endothelial surface facing upward. After preparation, the samples were placed in HBSS buffer supplemented with 1% FBS, 1% Pen/Strep and 5 mM glucose and directly used in AFM nanoindentation experiments.

### Confocal imaging

Aorta patches were prepared as described in the section *Aorta sample preparation*. Next, the aorta patches were fixed with 1.5% formaldehyde for 10 min. The samples were then washed 3 times in PBS and incubated with a solution of blocking peptide for 30 min. The samples were then gently washed in PBS and incubated with vWF Antibody (Santa Cruz Biotechnology sc-53466, 1:50) for 1 h at RT. Afterwards, the ECs were gently rinsed with PBS and incubated with m-IgGκ BP-CFL 594 (Santa Cruz Biotechnology). Additionally, cell nuclei were stained by Hoechst (ThermoFisher). Images were acquired with a Zeiss LSM710 confocal microscope with 40× 1.4 NA PlanApo objective lens and using the Zen 2012 Black Edition software supplied by the manufacturer.

### Acquisition of AFM indentation curves

AFM nanoindentation experiments were performed with a NanoWizard III system (JPK, Germany). All measurements were performed on unfixed aorta samples immersed in HBSS solution at room temperature. A spherical colloidal probe with a nominal diameter of 4.5 µm was attached to the cantilever (NovaScan, USA), which had a nominal spring constant of 0.02 N/m.

For each aorta sample, spatial maps of indentation curves were recorded at many random positions of the sample. Typically, each region-of-interest (ROI) consisted of 6 × 6 curves that were recorded on a 20 µm × 20 µm grid. The corresponding step size of approx. 3 µm was chosen to be slightly smaller than the probe diameter. For selected data presented in the Results, larger 18 × 18 maps were stitched from nine smaller ROIs to visualize the spatial variations in the nanomechanical parameters. The indentation curves were recorded for a maximal loading force of 0.5 nN at a velocity of 0.9 µm/s. In the whole experiment, nearly 10,000 indentation curves were collected and analysed.

### Statistical analysis

Due to the non-normal distribution of the AFM data, all datasets were logarithmically transformed. Data that were classified as endothelium with and without the glycocalyx were analysed separately. Statistical significance was tested using a two-way ANOVA with age of the mice and mouse type, i.e., diabetic db/db or control db/+ mice, as independent variables. ANOVA was followed by Bonferroni multiple comparison tests. The means and SEMs of the logarithmically transformed data were then back-transformed using the procedure described by Limpert *et al*.^64,65^.

### Data Availability

The datasets generated during and/or analysed during the current study are available from the corresponding author on request.

## Electronic supplementary material

Supplementary Information
