# Supplementary material for: AFM-based detection of glycocalyx degradation and endothelial stiffening in the db/db mouse model of diabetes
Source: Sci Rep. 2017 Nov 21;7:15951. doi: 10.1038/s41598-017-16179-7 (PMC5698475; doi:10.1038/s41598-017-16179-7)
Supplement: Supplementary file 1 — Supplementary Information [file 41598_2017_16179_MOESM1_ESM.pdf]

SUPPLEMENTARY INFORMATION

AFM-based detection of glycocalyx degradation and endothelial stiffening in the db/db mouse model of diabetes

Marta Targosz-Korecka[1*], Magdalena Jaglarz[1], Katarzyna Malek-Zietek[1], Aleksandra Gregorius[2], Agnieszka Zakrzewska[2], Barbara Sitek[2], Zenon Rajfur[4], Stefan Chlopicki[2,3] and Marek Szymonski[1]

[1]Center for Nanometer-scale Science and Advanced Materials, NANOSAM, Faculty of Physics, Astronomy and Applied Computer Science, Jagiellonian University, Łojasiewicza 11, 30-348 Krakow, Poland

[2]Jagiellonian Centre for Experimental Therapeutics, JCET, Jagiellonian University, Bobrzyńskiego 14, 30-348 Krakow, Poland

[3]Chair of Pharmacology, Jagiellonian University Medical College, Grzegórzecka 16, 31-531 Krakow, Poland

[4]Department of Biosystems Physics, Faculty of Physics, Astronomy and Applied Computer Science, Jagiellonian University, Łojasiewicza 11, 30-348 Krakow, Poland

*e-mail: marta.targosz-korecka@uj.edu.pl

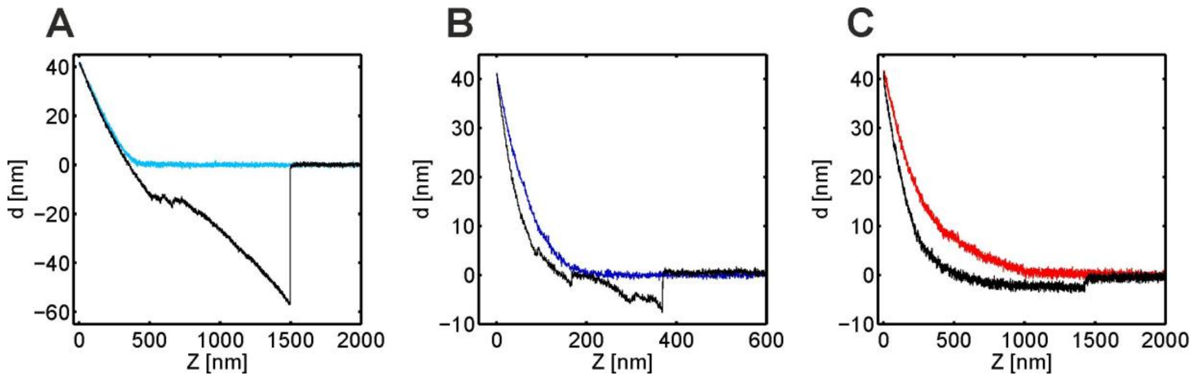

Figure S1. Examples of indentation curves recorded in the experiment together with retrace parts. **A-C** present the same curves as in Figure 1 of the paper. Retrace curves are shown as black lines.

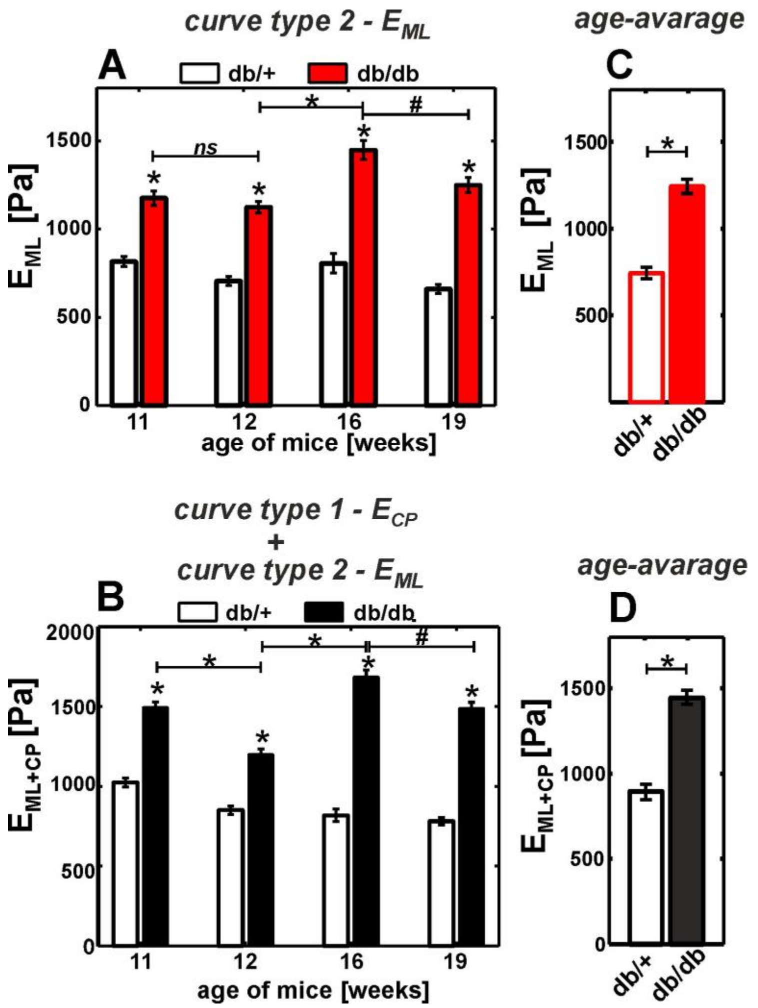

Figure S2. Extended analysis of the effect of endothelium stiffening in ex vivo mouse aorta. **(A)** Values of the elastic parameter derived from type 2 curves. **(B)** Values of the elastic parameter derived from combined analysis of type 1 and 2 curves. (C,D) Age-averaged values. Statistical significance was tested by two-way ANOVA followed by multiple-comparison Bonferroni tests. (*) $p<0.0001$. (ns) non-significant. For endothelium regions covered by the glycocalyx (type 2), the apparent elastic modulus of the endothelial layer was derived from the brush model (E_{ML}). For regions without the glycocalyx, the apparent elastic modulus of the endothelium was derived using a straightforward application of the Hertz model to the initial part of the indentation curves (E_{CP}). The quantity E_{ML+CP} reflects the net elastic modulus of the whole endothelium layer, which was calculated from a combined analysis of type 1 and 2 curves.
